# The Urinary Concentrations of Neutrophil Gelatinase-Associated Lipocalin, Cystatin C and Osteopontin in the Healthy Term and Stable Preterm Neonates: A Pilot Study

**DOI:** 10.3390/jcm12206512

**Published:** 2023-10-13

**Authors:** Monika Kamianowska, Aleksandra Kamianowska, Mateusz Maciejczyk, Anna Kurowska, Bożena Błażewicz, Agnieszka Maria Rogowska, Anna Wasilewska

**Affiliations:** 1 Department of Neonatology and Neonatal Intensive Care, Medical University of Bialystok, 15-174 Bialystok, Poland; 2 Department of Pediatrics and Nephrology, Medical University of Bialystok, 15-274 Bialystok, Poland; 3 Department of Hygiene, Epidemiology and Ergonomic, Medical University of Bialystok, 15-022 Bialystok, Poland

**Keywords:** neutrophil gelatinase-associated lipocalin (NGAL), cystatin C, osteopontin, kidney, neonates, biomarkers

## Abstract

Background: In neonates, the assessment of kidney function with serum creatinine is limited; therefore, more effective biomarkers are needed. Aim: The study aimed at analyzing the concentrations of renal biomarkers (osteopontin, cystatin C, and NGAL) in neonates. Material and Methods: The study included 80 term and 20 preterm neonates aged 28–33 weeks of gestation. Biomarkers were measured in urine. Term neonates’ urine was collected on the 1st day of life. Preterm neonates’ urine was collected on the 1st, 8th, 15th, 22nd day of life. Biomarkers’ concentrations were normalized to urinary creatinine (cr.) and presented as urinary biomarker/cr. ratios. Results: Median values of biomarker/creatine ratios in term and preterm neonates were the following: cystatin C/cr.: 7.26 and 439.49; osteopontin/cr.: 135.86 and 1633.37; NGAL/cr. in girls: 212.14 and 256.93; and NGAL/cr. in boys 27.123 and 65.29 ng/mg cr. In preterm neonates the cystatin C/cr. ratio was higher on the 1st than on the 8th day. The osteopontin/cr. ratio did not differ between the days. The NGAL/cr. ratio in girls was higher on the 8th than on the 22nd day, and in boys, the lowest was on the 22nd day. Conclusions: Prematurity in stable, Caucasian neonates might cause higher osteopontin and cystatin C excretion, but not NGAL. The excretion of NGAL and cystatin C, but not osteopontin, may change during first weeks of premature neonate’s life.

## 1. Introduction

Nowadays, serum creatinine is the gold standard for estimating the function of the kidneys and the glomerular filtration rate (GFR). However, the physiology of the neonatal period is an important factor in reducing the diagnostic value of this parameter [1]. The neonate’s serum creatinine levels reflect the maternal creatinine concentrations in blood serum. In neonates, the serum creatinine does not fully reflect the GFR; rather, it depends on various conditions [2,3,4]. It does not provide information on the type and location of the injury [5]. Because in the neonatal population the diagnosis of kidney injury and the assessment of kidney functions based on serum creatinine is limited, biomarkers other than serum creatinine have been investigated [5]. Understanding how these biomarkers work in healthy full-term and stable preterm babies is crucial for using them to evaluate renal function and injury in neonates. Therefore, this study aimed at analyzing the biomarkers of the renal function (osteopontin, cystatin C, and neutrophil gelatinase-associated lipocalin (NGAL)) in healthy term and stable preterm children. 

Osteopontin is produced in the kidneys, bones, and other organs. In the kidneys, it is synthetized in the descending thin limb of the Henle loop and some collecting ducts of the medulla. Then, it is secreted into the urine [6]. It helps cells regenerate and prevents calcium oxalate crystals, nitric oxide synthesis, and cell death. Its role in the kidneys is not fully understood. However, its role in tubulogenesis is suggested [7]. Osteopontin is important for the chemotaxis, recruitment, and adhesion of macrophages. An elevated expression of osteopontin and macrophage infiltration was described in both glomerular and interstitial nephritis [8]. The expression of osteopontin is elevated in urolithiasis, renal allograft dysfunction, and both acute and chronic kidney diseases [7].

Cystatin C, a polypeptide inhibitor of cysteine proteases, is synthetized in nucleated cells and then released in stable amounts to blood and extracellular fluid [9]. The serum concentration of cystatin C is widely used to assess the glomerular filtration rate [10]. Cystatin C is filtered in the glomerulus, then reabsorbed and metabolized in the cells of the proximal tubules. It is excreted only in small amounts in urine [11]. These amounts can be assessed in the urine. As cystatin C is almost totally reabsorbed in the proximal tubules, its higher levels in the urine may serve as a marker of tubular damage [9]. The urinary cystatin C is considered as a biomarker of the early phase of the kidney injury [12].

NGAL is a small molecule found in both plasma and urine, expressed at low levels in many tissues, including kidneys. It is expressed mostly in the distal part of the nephron (the thick part of the ascending limb of the Henle’s loop, the distal tubule and the collecting duct) [13,14]. Its expression is elevated in injured epithelia [15]. In kidneys, the synthesis of NGAL in cells increases shortly after renal injury but before an increase in serum creatinine [16]. NGAL induces bacteriostasis, has anti-apoptotic effects, and enhances proliferation of the renal tubules’ cells. These functions constitute the NGAL-mediated protection of the kidneys after injury [17,18]. Moreover, NGAL plays an important role in proliferating nephrons in the preterm neonates’ kidneys [19].

Considering the immaturity and ongoing nephrogenesis in the preterm kidneys, their susceptibility to injury resulting in inflammation, and the functions of the osteopontin (engaged in the tubulogenesis and the recruitment of macrophages), it was hypothesized that its levels will be higher in the preterm neonates. In terms of cystatin C, it was hypothesized that its levels will be higher in the urine of preterm neonates. Their proximal tubules are still immature, and it might cause the impaired reabsorption of cystatin C and higher amounts to be detected in the urine. Because in the kidneys the synthesis of NGAL in the cells increases shortly after renal injury, it was hypothesized that its values in the urine of preterm neonates will be higher than those in the urine of term neonates because their kidney are prone to injury. 

## 2. Patients and Methods

### 2.1. Patient Recruitment

A study was conducted at the Medical University of Bialystok’s Department of Neonatology and Neonatal Intensive Care. The local Bioethics Committee of the Medical University of Bialystok (protocol code APK.002.502.2021, date of approval: 16 December 2021) approved the study protocol. All procedures were conducted under the guidelines of the Declaration of Helsinki. The parents of all newborns were given detailed information about the study and what would happen after they received the results. 

The study observed 100 Caucasian West Slavic newborns born and hospitalized at a university hospital in Bialystok between January 2022 and January 2023.

Two groups were formed from the total of 100 neonates. The first group comprised 80 term neonates who were born between 37 and 42 weeks of pregnancy. The second group comprised 20 preterm children who were born between 28 and 33 weeks of pregnancy. Because the study was aimed at measuring values of the biomarkers of renal function in healthy neonates, it had strict inclusion criteria. We tried to exclude all possible postnatal and prenatal factors, which may affect the renal function. Only children who met the following inclusion criteria were included in the study: born in the Department of Perinatology of the University Clinical Hospital in Bialystok, parental written consent for the study, gestational age between 23 and 36 weeks for the preterm children and between 37 and 42 for the term children, and hospital stay of at least 22 days. 

The general exclusion criteria were divided into three groups. The first group included criteria related to neonatal factors, like genetic disorders, birth defects, and inborn diseases. The second group included criteria related to perinatal factors, like prenatal abnormalities. Group three had criteria regarding maternal health during pregnancy, like diseases, infections, and drug needs.

Neonates born between 23 and 36 weeks of pregnancy were excluded from the study if they met the specific exclusion criteria: first-minute Apgar score < 4, general condition described as severe, hospital infections, mechanical ventilation, abnormalities found in the ultrasound examination of the central nervous system (hyper-echoic zones around the lateral ventricles and first degree intraventricular bleeding were accepted), abnormalities in the ultrasound examination of the abdominal cavity, defects of the urinary tract (hydronephrosis, duplex kidney and duplex ureters, polycystic kidney disease, agenesis of the kidney, or other anatomical abnormalities), abnormalities in the laboratory tests (abnormal morphology and biochemistry), intrauterine growth retardation, exposure to nephrotoxic agents, the administration of drugs (we accepted the administration of mandatory drugs (vaccination against tuberculosis (BCG, Biomed Lublin SA, Lublin, Poland), vaccination against hepatitis B (Euvax B, LG Life Sciences, Warsaw, Poland), vitamin K (Konakion, Prima Infanzia, Roche Pharma AG, Basel, Schweiz or Kanavit, BB Pharma, Praha, Czech Republic), vitamin D3 (Devikap Polpharma, Starogard Gdanski, Poland)), methylxanthines (Peyona, Chiesi Farmaceutici, Parma, Italy), parenteral nutrition (Numeta G13 %E, Baxter, Warsaw, Poland), the supplementation of macro and micro elements (according with the standards)). 

Neonates born between 37 and 42 weeks of pregnancy were excluded from the study if they met the following specific exclusion criteria: first-minute Apgar score < 7, general condition described as average or severe, the administration of drugs (the administration of mandatory drugs was accepted (vaccination against tuberculosis (BCG, Biomed Lublin SA, Lublin, Poland), hepatitis B (Euvax B, LG Life Sciences, Warsaw, Poland), vitamin D3 (Devikap Polpharma, Starogard Gdanski, Poland), vitamin K (Konakion Prima Infanzia, Roche Pharma AG, Basel, Schweiz or Kanavit, BB Pharma, Praha, Czech Republic)).

The child’s participation in the study was terminated if the following conditions were met: the deterioration of the general condition, healthcare-associated infection, withdrawal of the parents’ written consent for the study.

**Figure 1:**
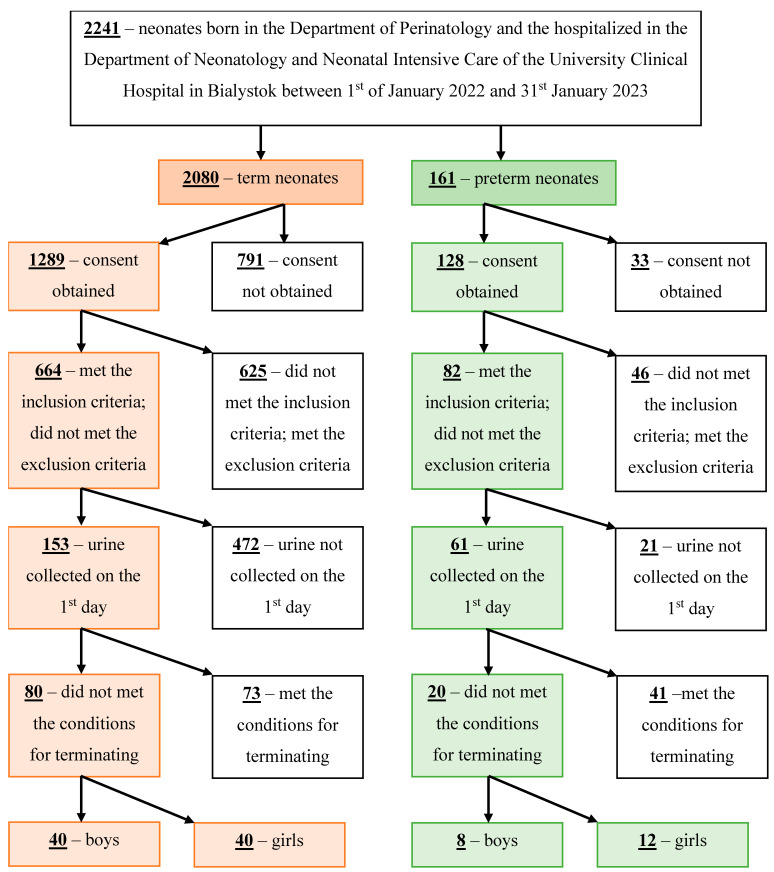
The algorithm of patients’ screening and creation of the final number of patients included in the study.

In Figure 1, we presented the algorithm of patients’ screening and the creation of the number of patients ultimately included in the study. 

### 2.2. Sample Collection

The urine of the term neonates was collected only once (1st day of life). The urine of the preterm neonates was collected four times (1st, 8th, 15th, and 22nd day of life). The urine samples were collected in a noninvasive way using sterile, single-use bags for the collection of the urine (ZARYS, Zabrze, Poland). All samples were centrifugated, stored in the refrigerator (at 4 °C) for 2 h, and then frozen and stored at −80 °C for 4 months. The samples were not thawed and frozen repeatedly. 

The blood of the preterm neonates was collected once (1st or 2nd day of life) as a part of a routine practice in the Department. The collection of venous blood was performed using S-Monovette 1.2 mL, Clotting Activator/Serum test tubes (Sarstedt AG & Co., Nümbrecht, Germany). Right after taking the samples, they performed blood tests (biochemistry and morphology). We assessed blood morphology (leucocytes, hemoglobin, hematocrit, and platelets) and biochemistry (C-reactive protein, procalcitonin, interleukin-6, urea, aspartate aminotransferase, alanine aminotransferase, bilirubin, protein, sodium, potassium, magnesium, and phosphorus).

### 2.3. Determination of Basic Blood and Urine Parameters 

We assessed the concentration of creatinine in blood serum and urine using Jaffé’s method. The unit of the concentration of creatinine was milligrams per deciliter (mg/dL).

We calculated the estimated glomerular filtration rate (eGFR) using the Schwartz formula. The formula was appropriate for preterm neonates ($eGFR=\frac{0.33\cdot length}{sCr}$, using length in centimeters (cm) (L) and the serum concentration of creatinine in milligrams per deciliter (mg/dL)) (sCr)). The unit of eGFR was milliliters per minute per 1.73 square meter (mL/min/1.73 m^2^).

The assessment of the blood morphology and biochemistry was performed in the Department of Laboratory Diagnostics at the University Clinical Hospital in Bialystok. We performed the tests as a part of the routine practice in the Laboratory.

### 2.4. Determination of the Urinary Concentration of Osteopontin, Cystatin C, and NGAL in the Urine

The biomarker’s concentrations were assessed in the urine. We used a kidney toxicity immunoassay called Bio-Plex Pro™ RBM Human Kidney Toxicity Panel 2 to measure biomarker concentrations. The assay had the following specifications:

- For the assessment of osteopontin: working range—3.80–2000.00 ng/mL; sensitivity—1.70 ng/mL; intra-assay coefficient of variation—6%; and inter-assay coefficient of variation—12%;
- For the assessment of cystatin C: working range—0.16–40.00 ng/mL; sensitivity—0.077 ng/mL; intra-assay coefficient of variation—3%; and inter-assay coefficient of variation—20%;
- For the assessment of NGAL: working range—0.062–34.00 ng/mL; sensitivity—0.052 ng/mL; intra-assay coefficient of variation—6%; and inter-assay coefficient of variation—12%.

The unit of the concentrations of all assessed biomarkers was nanograms per milliliter (ng/mL).

The concentrations of the biomarkers were measured in the Department of Hygiene, Epidemiology and Ergonomics of the Medical University of Bialystok. 

### 2.5. Determination of the Values of Osteopontin/cr., Cystatin C/cr., NGAL/cr. Ratios

Because of the potential confounding effect of the dilution of the urine, the concentrations of all assessed biomarkers were normalized to the concentration of creatinine in the urine. We expressed the results as ratios: osteopontin/cr., cystatin C/cr., and NGAL/cr. The unit of the received ratios was nanograms per milligram of creatinine (ng/mg cr.). 

### 2.6. Statistical Analysis

We analyzed the data using the Statistica 13.3 package (StatSoft, Cracow, Poland). Median and quartiles (Q1–Q3) were used to present continuous variables, while counts (percentage, %) were used to present discrete variables. We used the Shapiro–Wilk test to assess the distribution of the variables. The variables did not have a normal distribution. The Mann–Whitney U test as used to compare the continuous variables between the independent groups. Fisher’s exact test was used to check for any connection between two categorical variables. Spearman’s rank correlation coefficients were used to establish the strength and direction of the connections between biomarker concentrations, received ratios, and other variables. The Wilcoxon signed-rank test was used to compare the values of two dependent groups. We set the significance level at α = 0.05. The results were considered statistically significant at *p* < 0.05. 

In the group of term neonates, we determined the 95% confidence interval (the range of values including a population with 95% confidence).

## 3. Results

### 3.1. Characteristics of the Group Comprising Term Neonates

The first group comprised 80 healthy term neonates—40 girls and 40 boys (sex-matched, *p* > 0.05). The characteristics of the term neonates are presented in Table 1.

The term neonates were appropriate for gestational age, and their condition was assessed as good (first-minute Apgar score ≥ 8). Boys and girls had similar gestational age, birth weight, length, delivery type, and head circumference (*p* > 0.05 for each).

### 3.2. Characteristics of the Group Comprising Preterm Neonates

The second group comprised 20 preterm neonates—8 boys and 12 girls (sex-matched, *p* > 0.05). The characteristics of the preterm neonates group were presented in Table 2.

The gestational age of all premature neonates was between 28 and 33 weeks. Extremely preterm (aged <28 weeks of gestation) and late preterm (aged 34–36 weeks of gestation) were not included in the study because they met the exclusion criteria. The gestational age of the girls was significantly lower than the gestational age of the boys (*p* < 0.05). No significant difference was found in the proportion of vaginal- and cesarean-born children between boys and girls (*p* > 0.05). However, cesarean-born neonates dominated in both groups. Birth weight was significantly lower in the group of female neonates (*p* > 0.05). When compared to the term neonates, preterm neonates were characterized by lower birth weight, body length, and head circumference. 

The first-, third-, fifth- and tenth-minute Apgar scores were similar between the boys and the girls (*p* > 0.05). 

No significant differences in the parameters of the blood morphology and biochemistry were found between the boys and the girls (*p* > 0.05). The results were normal according to the hospital’s lab standards.

The preterm neonates had normal values of eGFR and the concentration of creatinine in serum and urine. No differences in the values of the above mentioned parameters were found between male and female neonates. A raising trend in the values of the eGFR on the following days was found. 

### 3.3. The Analysis of the Parameters of Renal Function

The detailed data on the values of the cystatin C/cr., osteopontin/cr. and NGAL/cr. ratios are presented in Table 3 (showing biomarkers on the 1st day of life in the term and preterm neonates) and in Table 4 (showing biomarkers on the 1st, 8th, 15th, and 22nd day of life in the preterm neonates).

### 3.4. The Analysis of the Concentrations of NGAL and the Values of NGAL/cr. Ratio

The values of the NGAL/cr. ratio on the 1st day of life did not differ between the term and preterm neonates, both in the girls and in the boys (*p* < 0.05 in each case).

Both in the term and the preterm neonates, the values of the NGAL/cr. ratio on the 1st day were significantly higher in the girls than in the boys (*p* < 0.05 in both cases).

In the preterm neonates the values of the NGAL/cr. ratio in the following days were higher in the girls than in the boys on each day of life (*p* < 0.05 on the 1st day, *p* < 0.01 on the 8th, 15th, and 22nd day of life). 

The values of the NGAL/cr. ratio did not differ between cesarean-born and vaginal born neonates, both in the term and the preterm children (*p* < 0.05 in each case) 

**Figure 2:**
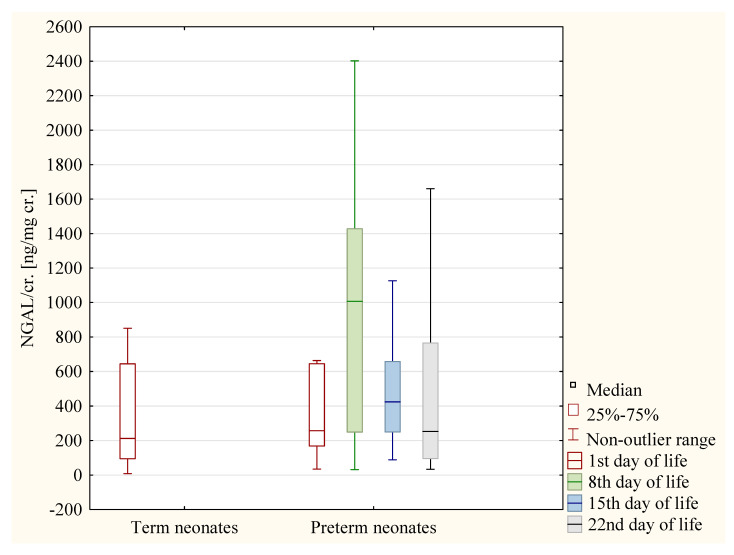
The values of NGAL/cr. ratio in term neonates and in preterm neonates on the following days of life—the group of girls.

**Figure 3:**
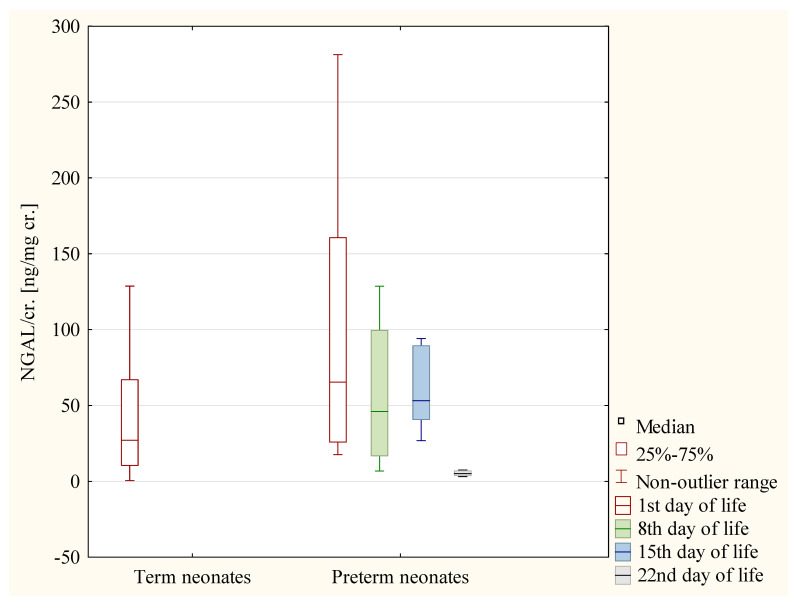
The values of NGAL/cr. ratio in term neonates and in preterm neonates on the following days of life—the group of boys.

In the preterm girls, the values of the NGAL/cr. ratio were significantly higher on the 8th day than on the 22nd day of life (*p* < 0.05) (Figure 2). In the preterm boys, the values of the NGAL/cr. ratio were significantly lower on the 22nd day of life than on the 1st, 8th, and 15th day of life (*p* < 0.05 in each case) (Figure 3). 

### 3.5. The Analysis of the Concentrations of Cystatin C and the Values of Cystatin C/cr. Ratio

The values of the cystatin C/cr. ratio on the 1st day of life were higher in the preterm neonates than in the term neonates (*p* < 0.01).

Both in the term and in the preterm neonates, no significant differences were found between the boys and the girls in the values of the cystatin C/cr. ratio on the 1st day.

In the preterm neonates, the values of the cystatin C/cr. ratio on the following days of life did not differ between the boys and the girls (*p* > 0.05).

In the term neonates, the values of the cystatin C/cr. ratio did not differ between cesarean- and vaginal-born neonates. In the preterm neonates, the values of the cystatin C/cr. ratio on the 1st day of life were lower in cesarean-born neonates (*p* < 0.01). The values of the cystatin C/cr. ratio on 8th, 15th, and 22nd day did not differ between cesarean- and vaginal-born ones.

**Figure 4:**
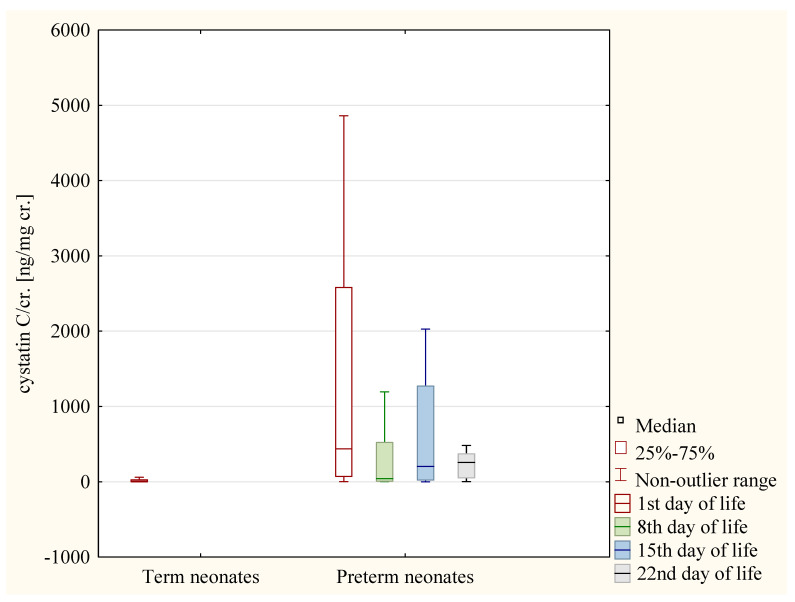
The values of cystatin C/cr. ratio in term neonates and in preterm neonates on the following days of life.

In the preterm neonates the values of the cystatin C/cr. ratio were significantly higher on the 1st day when compared to those on the 22nd day (*p* < 0.01) (Figure 4).

### 3.6. The Analysis of the Values of Osteopontin/cr. Ratio

The values of the osteopontin/cr. ratio on the 1st day of life was higher in the preterm neonates than in the term neonates (*p* < 0.01).

Both in the term and in the preterm neonates, no significant differences were found between boys and girls in the values of the osteopontin/cr. ratio on the 1st day.

In the preterm neonates, the values of the osteopontin/cr. ratio in the following days of life did not differ between male and female children (*p* > 0.05).

The values of the osteopontin/cr. ratio did not differ between cesarean-born and vaginal-born neonates both in the term and the preterm children (*p* < 0.05 in each case).

**Figure 5:**
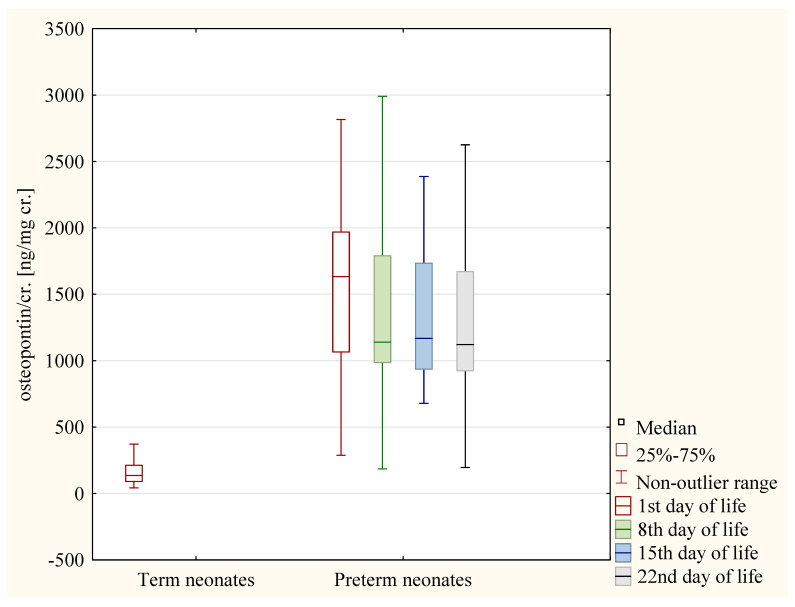
The values of osteopontin/cr. Ratio in term neonates and in preterm neonates on the following days of life.

In the preterm neonates, the values of osteopontin/cr. ratio were significantly higher on the 1st day when compared to the 2nd, 8th, and 22nd day (*p* < 0.01 in each case) (Figure 5). 

### 3.7. The Analysis of the Correlations

In the group of preterm children, a positive correlation was found between the following:

- The value of the osteopontin/cr. ratio and value of the cystatin C/cr. ratio (R = 0.42, *p* < 0.01);
- The value of the cystatin C/cr. ratio and the value of the NGAL/cr. ratio (R = 0.39, *p* < 0.01).

### 3.8. The Analysis of 95% CI for the Biomarkers of Renal Function in Term Neonates

We calculated the 95% CI for the values of the biomarker/cr. ratio in the group of term neonates. The detailed data are presented in Table 5

## 4. Discussion

In our study we showed the values of the cystatin C/cr., osteopontin/cr. and NGAL/cr. ratios in the term and preterm neonates. In Table 6, we presented the results of other studies available in the literature that concerned the biomarkers assessed in our study.

The serum cystatin C is widely used to assess the GFR; however, the role of urinary cystatin C in the assessment of renal injury is still uncertain [27]. When comparing our results with the results obtained by Li et al., it may be concluded that the values of the cystatin C/cr. ratio in healthy term neonates are similar than the values observed in the adult population. However, in stable preterm neonates, they are significantly higher than in healthy adults [29]. Our study found that the cystatin C/cr. ratio in healthy term neonates falls within the range proposed by Brott et al. for healthy adults. In healthy newborns, the cells of the proximal tubules can reabsorb cystatin C properly. A higher cystatin C/cr. ratio in preterm babies may indicate poor reabsorption by tubular cells due to immature or damaged proximal tubules. The study found that the kidneys of the preterm neonates did not show any signs of injury, but the results suggest that the kidneys may not have fully developed yet or there may be some hidden damage. According to Khosravi et al. the measurement of urinary cystatin C may be an early sensitive method to diagnose kidney injury in neonates [27]. According to Barbati et al. the concentration of cystatin C correlated negatively with the volume of the kidneys [25]. Preterm infants have fewer nephrons and renal proximal tubules, so their cystatin C levels may be higher. We did not found any significant differences in the values of the cystatin C/cr. ratio between boys and girls. Because serum cystatin C levels are also gender-independent, it can be concluded that changes in cystatin C levels in the urine are only because of changes in the proximal tubules’ function [38].

When comparing our results with the results obtained by Wasilewska et al., it may be concluded that the values of the osteopontin/cr. ratio in healthy term neonates are similar to its values in healthy children. Contrary, they are significantly higher in stable preterm neonates than in healthy children [31]. Our values of the osteopontin/cr. ratio in stable preterm neonates were consistent with the results obtained by Miklaszewska et al. [33]. We did not find any significant differences between male and female children in the values of the osteopontin/cr. ratio. Contrary, Miklaszewska et al. showed that the were higher in boys than in girls [33]. It could be explained by the small group of neonates examined in our study. According to Askenazi et al., preterm neonates with AKI have higher concentration of osteopontin in the urine than neonates with normal renal functions [21]. However, the higher levels of osteopontin in preterm neonates assessed in our study more likely resulted from renal immaturity and susceptibility to subclinical injury. Considering the fact that the expression of osteopontin is elevated during inflammation and oxidative stress and that both processes are parts of the pathogenesis of both glomerular and interstitial nephritis, its elevated concentrations in the urine may be found in these conditions [39]. However, the prematurity of the kidneys may also lead to its higher levels in the urine because of impaired glomerular and tubular functions [33]. Also, osteopontin prevents tubular cells apoptosis and takes part in the tubular cells repair [40]. Elevated urine levels of osteopontin may be due to kidney repair or subclinical injury in premature kidneys, and not necessarily a sign of clinical renal damage. 

The values of the NGAL/cr. ratio in healthy term neonates obtained in our study were not consistent with the results obtained by other researchers. The studies reported both lower and higher values of these parameters [22,26,35,36]. These differences may be explained by different days of the urine samples’ collection, different measurement methods, different reagents and antibodies used in the assessment [41]. In the literature there are only few studies assessing the concentration of NGAL in the urine of preterm children. Comparing these studies with our research, it may be concluded that both the values obtained by De Mul et al. and by Suchojad et al. were consistent with our results [34,37]. The lack of significant differences between term and preterm neonates is an interesting finding. However, a similar phenomenon was found by DeFreitas et al. [26]. We hypothesized that the concentration of NGAL will be higher in the preterm neonates. However, because higher levels of NGAL can be found in the urine, especially after the acute injury of the distal part of the nephron, it may suggest that these parts were not subclinically injured in the assessed children [42]. Immature kidneys may not reabsorb NGAL enough, causing slightly high levels. We found significantly higher values of the NGAL/cr. ratio in girls than in boys. These results are consistent with other researchers’ findings [24,36,37,43]. The explanation of this finding is that the urine of female patients can be contaminated by vaginal secretions containing NGAL [44]. In utero, the epithelium of the neonate’s vagina is stimulated by maternal hormones. Female newborns experience a fast drop in hormone levels after birth due to the lack of stimulation from maternal hormones through the placenta. Because of that, discharge from the vagina can be observed [45]. And this discharge contains NGAL as part of the innate immune system [44]. 

Studying the biomarkers in premature newborns during their first 22 days of life revealed changes in the cystatin C/cr. ratio and osteopontin/cr. ratio levels, which decreased significantly (*p* < 0.01, *p* < 0.01). We also found that the values of the NGAL/cr. ratio in the group of boys were significantly lower on the 22nd day than on the 1st, 8th, and 15th day. In the group of girls the values of the NGAL/cr. ratio were significantly lower on the 22nd day than the 8th day.

Unfortunately, there are few studies on the changes in biomarker concentration in preterm neonate urine during the first few days of life. There is a possible explanation for the decrease in the urinary concentrations of the assessed biomarkers (cystatin C, NGAL and osteopontin) observed in our study. The ongoing nephrogenesis and maturation of the kidneys leads to both an improvement in the renal function and a decrease in the kidneys’ vulnerability to injury. These changes lead to a reduction in the expression of osteopontin involved in both repair and inflammation in response to injury. An increase in the number of nephrons, and thus the reabsorption of cystatin C, lead to a reduction in its concentration in the urine. Because of the important role of NGAL in proliferating nephrons of the kidney in preterm neonates, the progression of renal maturation may lead to the decrease in the urinary NGAL concentration [19]. The changes of the concentrations of NGAL in the urine may be influenced by the possible contamination of the urine samples by vaginal secretions containing NGAL as part of the innate immune system, and the amount lowers over time [44]. 

## 5. Limitations

We view this study as an important pilot study. We evaluated a “special group” of babies who could be considered “healthy” among preterm infants. Other studies on kids without kidney damage include premature babies born from complex pregnancies, those treated with harmful drugs or having severe health issues. Creating our specific research group was difficult, therefore the size of the group of premature infants is relatively small. Nevertheless, the results of this pilot are very important to us from a clinical point of view. When taking care of premature babies, we look for typical values based on their age and week of life, even within the first day. The obtained results indicate that it is worth planning a long-term and preferably multicenter clinical study of renal biomarkers that considers at least the first weeks of the children’s life.

In this study, the urinary biomarkers were reported as normalized ratio to urinary creatinine concentration This normalization is used to avoid the confounding effect of the dilution of the urine. It is thought that creatinine is excreted at a constant and normal rate across and within individuals, which makes it a good factor for normalization. However, even a small change in the creatinine excretion can create a big change in the final value of the biomarker/cr. ratio. Urinary creatinine levels differ based on gender, age, weight, diet, individual variations, and changes in kidney function. In this case, understanding the relative effect of normalization on urinary creatinine should precede the analysis of urinary biomarker measurements [46,47]. 

Preterm infants, because of their immaturity, have widely varying renal function depending on the number of weeks. In fact, neonates who took part in the study were between 28 and 33 weeks of gestation age. We did not study extremely premature newborns (less than 28 weeks) because they require specialized care with antibiotics and other drugs that can harm their kidneys. We also did not include late preterm neonates (34–36 weeks), as they usually do not stay in the hospital for 4 weeks, which was an inclusion criterium to this study. In this case, significantly younger and older children were not included in the study, making the studied group less diverse and more reliable.

This study was aimed at assessing the urinary levels of the biomarkers. To understand how the body processes them, we should measure both their urine and serum levels together. 

We did not calculate the eGFR in term neonates. We do not have to take blood samples from healthy newborns in our department to test their creatinine levels. 

This preliminary study should be explored in larger studies. It seems valuable to conduct similar studies in various clinical conditions. Understanding how NGAL, osteopontin, and cystatin C are excreted is crucial for future biomarker qualification. 

## 6. Conclusions

In stable Caucasian West Slavic neonates, prematurity may be associated with the higher excretion of osteopontin and cystatin C, but not NGAL. The excretion of NGAL and cystatin C but not osteopontin may change during the first weeks of the premature neonate’s life. The female gender may be associated with the higher excretion of NGAL. The excretion of cystatin C and osteopontin may not depend on sex. 

## Figures and Tables

**Table 1:** The term neonates—the characteristics of the term neonates.

	All Term Neonates (N = 80)	Girls (N = 40)	Boys (N = 40)	*p*
	Median (Q1–Q3)			
Gestational age (weeks)	39 (38–40)	39 (39–40)	39 (37–40)	0.89
Vaginal delivery/ Cesarean delivery	41/39	22/18	19/21	0.65
Anthropometric data				
Birth weight (g)	3345 (3085–3755)	3355 (3125–3805)	3320 (2990–3710)	0.66
Birth weight (10th–50th percentile/ 51st–90th percentile)	43/37	21/19	22/18	1.00
Body length (cm)	55 (54–57)	55 (54–56)	56 (54–57)	0.49
Head circumference (cm)	34 (34–35)	34 (34–35)	35 (34–35)	0.82

*p*—comparison of boys and girls.

**Table 2:** The preterm neonates—the characteristics of the preterm neonates.

	All Preterm Neonates (N = 20)	Girls (N = 12)	Boys (N = 8)	*p*
	Median (Q1–Q3)			
Gestational age (weeks)	30.5 (29.5–32.5)	30.0 (28.5–31.5)	31.0 (30.5–33.0)	0.05
Vaginal delivery/Cesarean delivery	6/14	4/8	2/6	1.00
Anthropometric data				
Birth weight (g)	1600 (1220–1875)	1305 (1200–1680)	1875 (1600–2055)	0.02
Head circumference (cm)	29.0 (27.5–30.5)	29.0 (26.5–30.5)	30.0 (29.0–32.0)	0.10
Body length (cm)	45.0 (38.5–47.0)	41.0 (37.5–46.5)	46.0 (45.0–47.0)	0.21

*p*—comparison of boys and girls.

**Table 3:** The biomarkers of kidney function and injury in the term—the 1st day of life.

Term Neonates				
Parameters	All Neonates (N = 80)	Girls (N = 40)	Boys (N = 40)	*p*
	Median (Q1–Q3)			
Cystatin C/cr. (ng/mg cr.)	7.26 (1.08–27.83)	8.37 (1.60–30.29)	4.31 (0.87–26.26)	0.55
Osteopontin/cr. (ng/mg cr.)	135.86 (90.82–212.81)	132.97 (86.57–216.83)	138.79 (97.58–211.53)	0.82
NGAL/cr. (ng/mg cr.)	61.48 (16.09–212.14)	212.14 (94.74–643.90)	27.12 (10.52–66.88)	0.00

*p*—Comparison of boys and girls; NGAL—neutrophil gelatinase-associated lipocalin.

**Table 4:** The biomarkers of kidney function and injury in the preterm neonates—the 1st, 8th, 15th, and 22nd day of life.

	All Preterm Neonates (N = 20)	Girls (N = 12)	Boys (N = 8)	*p*
	Median (Q1–Q3)			
1st day of life				
Cystatin C/cr. (ng/mg cr.)	439.49 (71.68–2580.43)	701.02 (120.41–2906.65)	399.75 (9.89–1630.24)	0.34
Osteopontin/cr. (ng/mg cr.)	1633.37 (1065.31–1969.01)	1749.04 (133.66–2014.50)	1396.72 (654.99–1938.75)	0.46
NGAL/cr. (ng/mg cr.)	194.59 (65.29–277.70)	256.93 (168.13–644.88)	65.29 (25.98–160.54)	0.02
8th day of life				
Cystatin C/cr. (ng/mg cr.)	41.94 (7.17–523.09)	32.76 (5.50–523.09)	72.21 (27.39–696.83)	0.67
Osteopontin/cr. (ng/mg cr.)	1138.78 (985.50–1790.06)	1334.29 (1033.66–2149.22)	1100.65 (922.57–1318.56)	0.23
NGAL/cr. (ng/mg cr.)	215.94 (37.66–1051.51)	1006.35 (249.14–1427.86)	45.96 (16.85–99.49)	0.00
15th day of life				
Cystatin C/cr. (ng/mg cr.)	205.52 (24.52–1270.72)	205.52 (24.52–1562.29)	257.02 (62.10–882.56)	0.73
Osteopontin/cr. (ng/mg cr.)	1167.98 (935.20–1733.24)	1345.531 (1074.78–2086.85)	989.98 (839.99–1413.03)	0.08
NGAL/cr. (ng/mg cr.)	231.59 (69.57–470.99)	424.61 (249.96–657.23)	53.16 (40.75–89.28)	0.00
22nd day of life				
Cystatin C/cr. (ng/mg cr.)	258.31 (53.25–372.734)	233.20 (27.80–428.78)	258.31 (156.58–327.42)	0.96
Osteopontin/cr. (ng/mg cr.)	1121.38 (923.11–1670.59)	974.93 (168.13–644.88)	1320.22 (1077.69–2033.19)	0.08
NGAL/cr. (ng/mg cr.)	59.60 (5.96–431.48)	253.09 (95.21–764.86)	5.13 (3.82–7.02)	0.01

*p*—Comparison of boys and girls; NGAL—neutrophil gelatinase-associated lipocalin.

**Table 5:** Mean and 95% CI for the concentration of the assessed biomarkers and the values of biomarker/cr. ratios.

	Mean	95% CI
Cystatin C/cr. (ng/mg cr.)	39.20	19.24–59.17
Osteopontin/cr. (ng/mg cr.)	195.44	147.92–242.77
NGAL/cr. (ng/mg cr.)—boys	58.74	26.97–90.50
NGAL/cr. (ng/mg cr.)—girls	1063.70	225.03–1902.37

95% CI—95% confidence interval.

**Table 6:** Results of studies concerning urinary cystatin C, osteopontin, and NGAL concentrations.

Authors	Year	Subjects	Results
Urinary Cystatin C			
Koyner et al. [20]	2008	Acute kidney injury in adults undergoing cardiac surgery	Before surgery: median cystatin C/cr. ratio in patients who developed AKI after surgery and in patients who did not was similar: 50 and 60 ng/mg cr. After surgery: median cystatin C/cr. ratio in patients without AKI (290 ng/mg cr.) and was lower than in patients with AKI (1300 ng/mg cr.) (*p* < 0.001).
Askenazi et al. [21]	2011	Very low birth weight neonates	Median concentration of cystatin C in neonates without AKI: 2150 ng/mL.
Sarafidis et al. [22]	2012	Asphyxiated neonates and healthy term neonates	Median cystatin C/cr. ratio in healthy neonates on the 1st, 3rd, and 10th day of life: 148, 169, and 140 ng/mg cr., respectively.
Brott et al. [23]	2014	Healthy term neonates	95% CI for the cystatin C/cr. ratio in healthy adults: 17.5–70.4 ng/mg cr.
Askenazi et al. [24]	2016	Very low birth weight neonates	Median cystatin C/cr. ratio in in neonates without AKI 7230 ng/mg cr.
Barbati et al. [25]	2016	Neonates with intrauterine growth retardation (IUGR)	Concentration of cystatin C in neonates with IUGR was significantly higher than in appropriate for gestational age children.
DeFreitas et al. [26]	2016	Preterm and term neonates	Cystatin C/cr. ratio in term neonates at birth (geometric mean, 95% CI): 210 (86, 513) ng/mg cr. Cystatin C/cr. ratio in preterm neonates at birth (geometric mean, 95% CI): 315 (195, 508) ng/mg cr. Cystatin C/cr. ratio did not change between birth and 3rd month of life.
Khosravi et al. [27]	2018	Neonates	The urinary cystatin C level could predict kidney injury and effectively discriminate kidney injury from normal kidney function (AUC = 0.868, 95% CI: 0.811–0.925, *p* < 0.001).
Sridahran et al. [28]	2021	Neonates receiving potentially nephrotoxic drugs	Median cystatin C/cr. ratio: 6.63 ng/mg cr.
Li et al. [29]	2022	Healthy adults	Median cystatin C/cr. ratio: 20.9 ng/mg cr.
Coskun et al. [30]	2022	Preterm neonates	Median concentration of cystatin C: on the 1st or 2nd day of life: 1600–9200 ng/mL; on the 7th day: 900–12,100 ng/mL; on the 14th day: 400–12,600 ng/mL; on the 21st day: 600–2000 ng/mL; on the 28th day: 0 ng/mL.
Urinary osteopontin			
Wasilewska et al. [31]	2011	Healthy children	Median osteopontin/cr. ratio: 68.84 ng/mg cr.
Askenazi et al. [21]	2011	Preterm neonates	Median concentration of osteopontin in neonates with AKI vs. in neonates with normal renal functions: 217 vs. 468 ng/mL.
Al-Malki [32]	2014	Diabetic patients with nephropathy	The urinary level of osteopontin has a good accuracy in distinguishing between patients with nephropathy and healthy controls (AUC = 0.73, sensitivity = 92.3% and specificity = 89.9%.
Brott et al. [23]	2014	Healthy adults	95% CI for the osteopontin/cr. ratio in healthy adults: 236–1459 ng/mg cr.
DeFreitas et al. [26]	2016	Preterm and term neonates	Osteopontin/cr. ratio in term neonates at birth (geometric mean, 95% CI): 377 (138, 1030) ng/mg cr. Osteopontin/cr. ratio in preterm neonates at birth (geometric mean, 95% CI): 670 (389, 1155) ng/mg cr. Osteopontin/cr. ratio decreased between birth and 3rd month of life.
Askenazi et al. [24]	2016	Very low birth weight neonates	Median osteopontin/cr. ratio in neonates without AKI: 4640 ng/mg cr.
Miklaszewska et al. [33]	2019	Premature children	Osteopontin/cr. ratio in LBW and VLBW neonates (geometric mean, 50%CI): in girls: 493.4 (234.4–1038.4) and 1478.9 (699.5–3126.8); in boys: 897.7 (518.9–1553.1) and 1754.8 (787.8–3908.8) ng/mg cr. Osteopontin/cr. ratio in LBW neonates was lower in the following 4 weeks of life, but the differences were not statistically significant.
Urinary NGAL			
Askenazi et al. [21]	2011	Very low birth weight neonates	Median concentration of NGAL in neonates without AKI vs. in neonates with AKI: 458 vs. 985 ng/mL.
Sarafidis et al. [22]	2012	Asphyxiated neonates and healthy term neonates	Median NGAL/cr. ratio in healthy neonates on the 1st, 3rd, and 10th day of life: 14.5, 25.7, and 8 ng/mg cr.
Suchojad et al. [34]	2015	Preterm neonates without AKI	Median concentration of urinary NGAL: 144 ng/mL).
Askenazi et al. [24]	2016	Very low birth weight neonates	Median NGAL/cr. ratio in neonates without AKI: 3170 ng/mg cr.
Chen et al. [35]	2016	Term and preterm neonates	Median concentration of NGAL in term neonates on the 3rd day of life: 88.1 ng/mL. Median concentrations of NGAL in premature neonates on the 3rd, 7th, 14th, and 21st day of life were: 41.52, 35.82, 43.79, and 30.85 ng/mL, respectively (*p* > 0.05).
DeFreitas et al. [26]	2016	Preterm and term neonates	NGAL/cr. ratio in term neonates at birth (geometric mean, 95% CI): 335 (135, 834) ng/mg cr. NGAL/cr. ratio in preterm neonates at birth (geometric mean, 95% CI): 568 (347, 929) ng/mg cr. NGAL/cr. ratio in term neonates and preterm neonates at birth did not differ significantly (*p* = 0.33). NGAL/cr. ratio decreased between birth and 3rd month of life.
Kamianowska et. al. [36]	2016	Term neonates, first 48 h of life	Higher median NGAL/cr. ratio was observed in girls than in boys: 37.06 and 19.36 ng/mg cr., respectively.
Miklaszewska et al. [33]	2019	Preterm neonates	NGAL/cr. ratio decreased in the following weeks of life in LBW, VLBW, and ELBW male neonates. For female neonates, a similar trend was observed only in VLBW children.
Sridahran et al. [28]	2021	Neonates receiving potentially nephrotoxic drugs	Median NGAL/cr. ratio: 38.45 ng/mg creatinine.
De Mul at. al [37]	2022	Preterm neonates without AKI	Median concentration of urinary NGAL: 122.8 ng/mL.

AKI—Acute kidney injury; NGAL—neutrophil gelatinase-associated lipocalin; LBW—low birth weight; VLBW—very low birth weight; ELBW—extremely low birth weight; cr.—creatinine.

## Data Availability

Not applicable.

## References

[1] Allegaert K., Smits A., Van Donge T., van den Anker J., Sarafidis K., Levtchenko E., Mekahli D. (2020). Renal Precision Medicine in Neonates and Acute Kidney Injury: How to Convert a Cloud of Creatinine Observations to Support Clinical Decisions. Front. Pediatr..

[2] Kastl J.T. (2017). Renal function in the fetus and the neonate—The creatinine enigma. Semin. Fetal Neonatal Med..

[3] Schreuder M.F., Bueters R.R., Allegaert K. (2014). The interplay between drugs and the kidney in premature neonates. Pediatr. Nephrol..

[4] Modi N., Hutton J.L. (1990). Urinary creatinine excretion and estimation of muscle mass in infants of 25–34 weeks gestation. Acta Paediatr. Scand..

[5] Kamianowska M., Szczepański M., Wasilewska A. (2019). Tubular and Glomerular Biomarkers of Acute Kidney Injury in Newborns. Curr. Drug Metab..

[6] Hudkins K.L., Giachelli C.M., Cui Y., Couser W.G., Johnson R.J., Alpers C.E. (1999). Osteopontin expression in fetal and mature human kidney. J. Am. Soc. Nephrol..

[7] Kaleta B. (2019). The role of osteopontin in kidney diseases. Inflamm. Res..

[8] Yu X.Q., Wu L.L., Huang X.R., Yang N., Gilbert R.E., Cooper M.E., Johnson R.J., Lai K.N., Lan H.Y. (2000). Osteopontin expression in progressive renal injury in remnant kidney: Role of angiotensin II. Kidney Int..

[9] Westhuyzen J. (2006). Cystatin C: A promising marker and predictor of im paired renal function. Ann. Clin. Lab. Sci..

[10] Ferguson T.W., Komenda P., Tangri N. (2015). Cystatin C as a biomarker for estimating glomerular filtration rate. Curr. Opin. Nephrol. Hypertens..

[11] Mussap M., Plebani M. (2004). Biochemistry and clinical role of human cystatin C. Crit. Rev. Clin. Lab. Sci..

[12] Park M.Y., Choi S.J., Kim J.K., Hwang S.D., Lee Y.W. (2013). Urinary cystatin C levels as a diagnostic and prognostic biomarker in patients with acute kidney injury. Nephrology.

[13] Marakala V. (2022). Neutrophil gelatinase-associated lipocalin (NGAL) in kidney injury—A systematic review. Clin. Chim. Acta.

[14] Schmidt-Ott K.M. (2011). Neutrophil gelatinase-associated lipocalin as a biomarker of acute kidney injury—Where do we stand today?. Nephrol. Dial. Transplant..

[15] Devarajan P., Ronco C., Bellomo R., Kellum J.A. (2007). Acute Kidney Injury. Contrib. Nephrol..

[16] Bolignano D., Donato V., Coppolino G., Campo S., Buemi A., Lacquaniti A., Buemi M. (2008). Neutrophil Gelatinase–Associated Lipocalin (NGAL) as a Marker of Kidney Damage. Am. J. Kidney Dis..

[17] Mori K., Lee H.T., Rapoport D., Drexler I.R., Foster K., Yang J., Schmidt-Ott K.M., Chen X., Li J.Y., Weiss S. (2005). Endocytic delivery of lipocalin-siderophore-iron complex rescues the kidney from ischemia-reperfusion injury. J. Clin. Investig..

[18] Mori K., Nakao K. (2007). Neutrophil gelatinase-associated lipocalin as the real-time indicator of active kidney damage. Kidney Int..

[19] Pan J.J., Sun Z.Y., Zhou X.Y., Hu Y.H., Cheng R., Chen X.Q., Yang Y. (2018). Is neutrophil gelatinase-associated lipocalin a good diagnostic marker for renal injury in asphyxiated preterm infants?. J. Res. Med. Sci..

[20] Koyner J.L., Bennett M.R., Worcester E.M., Ma Q., Raman J., Jeevanandam V., Kasza K.E., O’Connor M.F., Konczal D.J., Trevino S. (2008). Urinary cystatin C as an early biomarker of acute kidney injury following adult cardiothoracic surgery. Kidney Int..

[21] Askenazi D.J., Montesanti A., Hunley H., Koralkar R., Pawar P., Shuaib F., Liwo A., Devarajan P., Ambalavanan N. (2011). Urine biomarkers predict acute kidney injury and mortality in very low birth weight infants. J. Pediatr..

[22] Sarafidis K., Tsepkentzi E., Agakidou E., Diamanti E., Taparkou A., Soubasi V., Papachristou F., Drossou V. (2012). Serum and urine acute kidney injury biomarkers in asphyxiated neonates. Pediatr. Nephrol..

[23] Brott D., Adler S.H., Arani R., Lovick S.C., Pinches M., Furlong S.T. (2014). Characterization of renal biomarkers for use in clinical trials: Biomarker evaluation in healthy volunteers. Drug Des. Dev. Ther..

[24] Askenazi D.J., Koralkar R., Patil N., Halloran B., Ambalavanan N., Griffin R. (2016). Acute Kidney Injury Urine Biomarkers in Very Low-Birth-Weight Infants. Clin. J. Am. Soc. Nephrol..

[25] Barbati A., Cappuccini B., Aisa M.C., Grasselli C., Zamarra M., Bini V., Bellomo G., Orlacchio A., Di Renzo G.C. (2016). Increased Urinary Cystatin-C Levels Correlate with Reduced Renal Volumes in Neonates with Intrauterine Growth Restriction. Neonatology.

[26] DeFreitas M.J., Seeherunvong W., Katsoufis C.P., RamachandraRao S., Duara S., Yasin S., Zilleruelo G., Rodriguez M.M., Abitbol C.L. (2016). Longitudinal patterns of urine biomarkers in infants across gestational ages. Pediatr. Nephrol..

[27] Khosravi N., Zadkarami M., Chobdar F., Hoseini R., Khalesi N., Panahi P., Karimi A. (2018). The Value of Urinary Cystatin C Level to Predict Neonatal Kidney Injury. Curr. Pharm. Des..

[28] Sridharan K., Al Jufairi M., Al Segai O., Al Ansari E., Ahmed H.H., Shaban G.H., Malalla Z., Al Marzooq R., Al Madhoob A., Tabbara K.S. (2021). Biomarkers in neonates receiving potential nephrotoxic drugs. Eur. Rev. Med. Pharmacol. Sci..

[29] Li B., Zamzam A., Syed M.H., Jahanpour N., Jain S., Abdin R., Qadura M. (2022). Urinary Cystatin C Has Prognostic Value in Peripheral Artery Disease. Biomolecules.

[30] Coskun Y., Demirel O.U., Bayram T., Akman I., Hacihamdioglu D.O. (2022). Estimating glomerular filtration rate via cystatin-C in preterm infants: A comparative analysis. Paediatr. Indones..

[31] Wasilewska A., Taranta-Janusz K., Kuroczycka-Saniutycz E., Zwierz W.Z. (2011). Urinary OPN excretion in children with glomerular proteinuria. Adv. Med. Sci..

[32] Al-Malki A.L. (2014). Assessment of urinary osteopontin in association with podocyte for early predication of nephropathy in diabetic patients. Dis. Markers.

[33] Miklaszewska M., Korohoda P., Drożdż D., Zachwieja K., Tomasik T., Moczulska A., Korzeniecka-Kozerska A., Kwinta P. (2019). eGFR values and selected renal urine biomarkers in preterm neonates with uncomplicated clinical course. Adv. Clin. Exp. Med..

[34] Suchojad A., Tarko A., Smertka M., Majcherczyk M., Brzozowska A., Wroblewska J., Maruniak-Chudek I. (2015). Factors limiting usefulness of serum and urinary NGAL as a marker of acute kidney injury in preterm newborns. Ren. Fail..

[35] Chen C.-N., Chou C.-H., Jeng S.-F., Tsai I.-J., Chen P.-C., Chen C.-Y., Chou H.-C., Tsao P.-N., Hsieh W.-S. (2016). Urinary neutrophil gelatinase-associated lipocalin levels in neonates. Pediatr. Neonatol..

[36] Kamianowska M., Wasilewska A., Szczepański M., Kulikowska E., Bebko B., Koput A. (2016). Health term-born girls had higher levels of urine neutrophil gelatinase-associated lipocalin than boys during the first postnatal days. Acta Paediatr..

[37] De Mul A., Parvex P., Wilhelm-Bals A. (2022). Neutrophil gelatinase-associated lipocalin distribution in preterm newborns without acute kidney injury as defined by a reference method. J. Matern. Fetal Neonatal Med..

[38] Galteau M.M., Guyon M., Gueguen R., Siest G. (2001). Determination of serum cystatin C: Biological variation and reference values. Clin. Chem. Lab. Med..

[39] Trostel J., Truong L.D., Roncal-Jimenez C., Miyazaki M., Miyazaki-Anzai S., Kuwabara M., McMahan R., Andres-Hernando A., Sato Y., Jensen T. (2018). Different effects of global osteopontin and macrophage osteopontin in glomerular injury. Am. J. Physiol. Ren. Physiol..

[40] Xie Y., Sakatsume M., Nishi S., Narita I., Arakawa M., Gejyo F. (2001). Expression, roles, receptors, and regulation of osteopontin in the kidney. Kidney Int..

[41] Krzeminska E., Wyczalkowska-Tomasik A., Korytowskam N., Paczekm L. (2016). Comparison of Two Methods for Determination of NGAL Levels in Urine: ELISA and CMIA. J. Clin. Lab. Anal..

[42] Parravicini E., Nemerofsky S.L., Michelson K.A., Huynh T.K., Sise M.E., Bateman D.A., Lorenz J.M., Barasch J.M. (2010). Urinary neutrophil gelatinase-associated lipocalin is a promising biomarker for late onset culture-positive sepsis in very low birth weight infants. Pediatr. Res..

[43] Haase M., Bellomo R., Devarajan P., Schlattmann P., Haase-Fielitz A., NGAL Meta-Analysis Investigator Group (2009). NGAL Meta-analysis Investigator Group. Accuracy of neutrophil gelatinase-associated lipocalin (NGAL) in diagnosis and prognosis in acute kidney injury: A systematic review and meta-analysis. Am. J. Kidney Dis..

[44] Beghini J., Giraldo P.C., Linhares I.M., Ledger W.J., Witkin S.S. (2015). Neutrophil Gelatinase-Associated Lipocalin Concentration in Vaginal Fluid: Relation to Bacterial Vaginosis and Vulvovaginal Candidiasis. Reprod. Sci..

[45] Wróblewska-Seniuk K., Jarząbek-Bielecka G., Kędzia W. (2021). Gynecological Problems in Newborns and Infants. J. Clin. Med..

[46] Wagner B.D., Accurso F.J., Laguna T.A. (2010). The applicability of urinary creatinine as a method of specimen normalization in the cystic fibrosis population. J. Cyst. Fibros..

[47] Waikar S.S., Sabbisetti V.S., Bonventre J.V. (2010). Normalization of urinary biomarkers to creatinine during changes in glomerular filtration rate. Kidney Int..

